# Linking cell cycle to hematopoietic stem cell fate decisions

**DOI:** 10.3389/fcell.2023.1231735

**Published:** 2023-08-14

**Authors:** Sydney Treichel, Marie-Dominique Filippi

**Affiliations:** ^1^ Division of Experimental Hematology and Cancer Biology, Department of Pediatrics, Cincinnati Children’s Hospital Research Foundation, Cincinnati, OH, United States; ^2^ University of Cincinnati College of Medicine, Cincinnati, OH, United States; ^3^ Molecular and Development Biology Graduate Program, University of Cincinnati College of Medicine, Cincinnati, OH, United States

**Keywords:** hematopoietic stem cells, cell cycle, fate decision, differentiation, asymmetric division

## Abstract

Hematopoietic stem cells (HSCs) have the properties to self-renew and/or differentiate into any blood cell lineages. In order to balance the maintenance of the stem cell pool with supporting mature blood cell production, the fate decisions to self-renew or to commit to differentiation must be tightly controlled, as dysregulation of this process can lead to bone marrow failure or leukemogenesis. The contribution of the cell cycle to cell fate decisions has been well established in numerous types of stem cells, including pluripotent stem cells. Cell cycle length is an integral component of hematopoietic stem cell fate. Hematopoietic stem cells must remain quiescent to prevent premature replicative exhaustion. Yet, hematopoietic stem cells must be activated into cycle in order to produce daughter cells that will either retain stem cell properties or commit to differentiation. How the cell cycle contributes to hematopoietic stem cell fate decisions is emerging from recent studies. Hematopoietic stem cell functions can be stratified based on cell cycle kinetics and divisional history, suggesting a link between Hematopoietic stem cells activity and cell cycle length. Hematopoietic stem cell fate decisions are also regulated by asymmetric cell divisions and recent studies have implicated metabolic and organelle activity in regulating hematopoietic stem cell fate. In this review, we discuss the current understanding of the mechanisms underlying hematopoietic stem cell fate decisions and how they are linked to the cell cycle.

## Introduction

Hematopoietic stem cells (HSCs) are defined by their multipotency or ability to differentiate into all types of blood cells, as well as their ability to self-renew and maintain the stem cell pool to support mature blood cell production. They reside at the top of the hierarchy as they differentiate into increasingly restricted progenitors to produce mature blood cells of all lineages (Seita and Weissman, 2010). The HSC pool is heterogeneous and can be classified into subsets that have distinct functional behaviors in terms of the duration (Long-term (LT), Intermediate (IT), or Short-Term (ST) HSC), the amplitude (high or low), and the nature (myeloid or lymphoid-bias) of their cellular output (Haas et al., 2018). The decisions to self-renew or to commit to differentiation must be tightly regulated, as loss of self-renewal leads to depletion of the stem cell pool and eventually bone marrow failure, but failure to differentiate can lead to leukemogenesis.

HSCs are mostly in a quiescent or dormant state in order to protect them from oxidative and replicative stress. Indeed, the self-renewal activity of adult HSCs is not unlimited and accumulating evidence indicates that HSCs progressively lose their long-term regenerative potential with each round of division *in vivo* (Wilson et al., 2008; Yamamoto et al., 2013; Qiu et al., 2014; Bernitz et al., 2016; Hinge et al., 2017). The quiescent state is thought to support the stemness of HSCs through protection from functional exhaustion and is regulated by both cell-intrinsic factors such as transcription factors, cell cycle regulators, metabolic activity, and epigenetic factors, as well as extrinsic factors such as signals from the HSC microenvironment, or niche. Yet, the decision to self-renew or to commit to differentiation inherently occurs when HSC are actively cycling, during which HSCs decide whether to continue dividing and thus commit to differentiation, or to re-enter quiescence and maintain some stemness characteristics (G_0_). Cells that will divide enter a growth or gap phase (G_1_), followed by replication of DNA in S phase, and a second growth phase (G_2_) in preparation for mitosis (Blagosklonny and Pardee, 2002). Progression through these phases is heavily regulated by cell signaling driven by the activity of specific cyclin proteins and cyclin-dependent kinases (CDKs) (Blagosklonny and Pardee, 2002). Interestingly, the stratification of HSCs by their repopulation capacity is correlated with distinct division kinetics with LT-HSCs possessing longer quiescent exit time than HSCs with short-term repopulation activity (Cheshier et al., 1999; Passegué et al., 2005; Wilson et al., 2008; Benveniste et al., 2010; Laurenti et al., 2015). Other studies have shown that HSC fate is controlled during the cell cycle and can be altered by signaling, metabolic pathways, or organelle functions (Kent et al., 2008; Ito et al., 2012; Loeffler et al., 2019; Hinge et al., 2020; Loeffler et al., 2022). Understanding the mechanisms underlying lineage commitment and fate decisions of HSCs during cell division will allow for a better understanding of physiological HSC function and differentiation, as well as dysregulation of HSC activity in the context of aging and hematological diseases. It will also allow for a better understanding of how to expand HSCs *ex vivo*, which has been a long-standing challenge.

In this review, we will summarize evidence linking cell cycle and fate decisions in hematopoietic stem and progenitor populations. We also review the mechanisms behind HSC fate decisions during active cycling. Most studies referred in this review were done in the murine system. Studies done in any other cell types, including human, will be specifically stated as they are reviewed. In addition, a number of different surface markers are used to identify murine HSCs for experimental studies. HSCs are commonly identified as Lineage-Sca1+cKit+CD48^−^CD150+ (or LSK-SLAM) (Kiel et al., 2005) which contains both LT-HSC and multipotent stem and progenitor cells. This population can then be further enriched for LT-HSCs by their CD34 and CD135/Flk2/Flt3 negativity (Osawa et al., 1996; Adolfsson et al., 2001), as well as Endothelial protein C receptor (EPCR or CD201) positivity (Kent et al., 2009; Kent et al., 2016). HSCs defined by different combinations of markers may behave similarly; this should therefore be considered when interpreting these studies.

## General evidence linking cell cycle to fate decisions

The link between cell cycle and cell fate has been well established in other stem cell populations (Dalton, 2015). Work in pluripotent embryonic stem cells (ESCs) has shown that when exposed to differentiation cues, initiation of cell fate commitment is linked to the cell cycle, specifically in the G_1_ phase, where cells are primed to activate developmental genes to initiate fate decisions (Dalton, 2015). While many of the molecular mechanisms underlying this phenomenon remain unclear, it is understood that cell cycle-dependent mechanisms regulate the ability to respond to differentiation cues. The mechanism behind these responses involves the integration of external signals with cell cycle regulation and the alteration of the chromatin landscape (Ma et al., 2015). Regulation of histone biogenesis, post-translational modifications (PTMs) of histone proteins, and covalent nucleotide modification in DNA are all mechanisms by which transcription and ultimately, cell fate, can be altered. As a cell progresses through G_1_ phase, changes in epigenetic and chromatin architecture create a favorable environment for the activation of development programs. In pluripotent stem cells, an increase in 5-hydroxymethylcytosine (5hmC) at developmental genes can be observed in G_1_, suggesting a permissive chromatin state for cells to respond to differentiation cues in G_1_ (Singh et al., 2013).

Other studies used the Fucci system, which allows for monitoring of cell cycle progression without relying on synchronizing drugs or fixation, to show that cell signaling pathways regulate developmental gene activation in a cell-cycle dependent manner (Pauklin and Vallier, 2013). The major effector of TGFβ/Activin/Nodal signaling, Smad2/3, is able to bind and activate specific developmental genes during early G_1_ phase in ESCs. However, in later G_1_, its translocation to the nucleus is limited by Cyclin D activity, allowing cells to adopt a different germ layer fate. However, whether similar cell cycle-dependent mechanisms are utilized by HSCs to regulate lineage commitment remains unclear.

## Cell cycle regulation and hematopoietic progenitor fate

In the hematopoietic system, a link between cell cycle regulation and fate decisions is demonstrated in progenitor lineage commitment. Lineage differentiation occurs in a stepwise manner during which cells traverse through different cell states, including bipotent states where the cells can choose to differentiate into one lineage or the other. Such binary choice exists between the myeloid and lymphoid lineage. It is known that myeloid differentiation is associated with high levels of the transcription factor PU.1, whereas downregulation of PU.1 is necessary for lymphoid cell fate. Interestingly, PU.1 controls these cell fate decisions via positive feedback with the cell cycle (Kueh et al., 2013). It was found that while B cells decrease PU.1 levels by reducing its transcription, macrophages lengthen their cell cycles and allow PU.1 to accumulate during differentiation.

**FIGURE 1:**
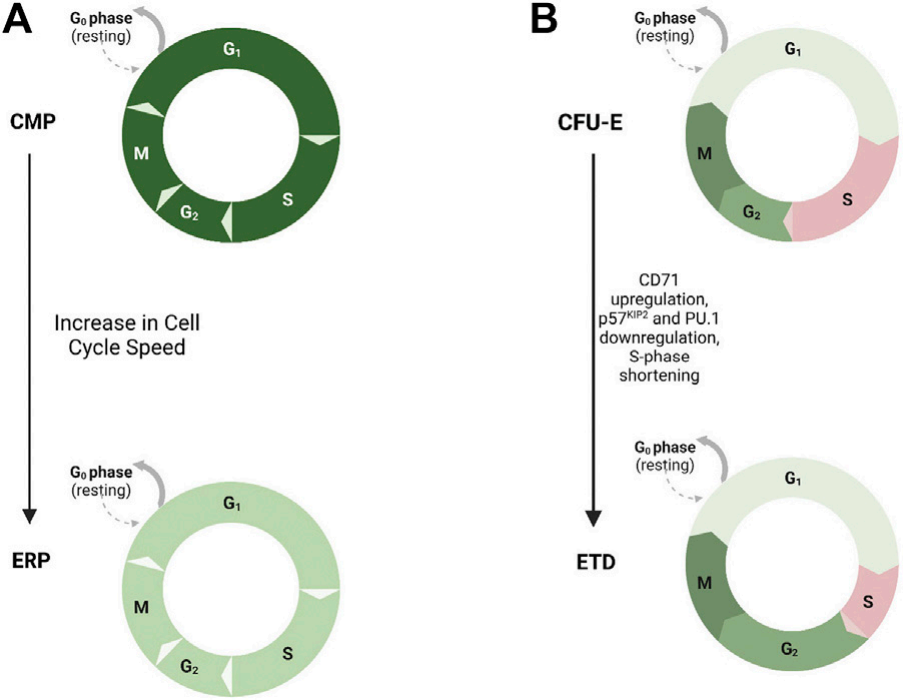
Cell Cycle Length and Hematopoietic Progenitor Fate. Model demonstrating the role of cell cycle speed in erythroid differentiation. **(A)** Increased cell cycle speed has been shown to drive differentiation of erythroid progenitors (ERPs--light green, faster speed) from common myeloid progenitors (CMPs--dark green, slower speed). **(B)** Shortening of S-phase duration, upregulation of CD71, and downregulation of CDK inhibitor p57^KIP2^ and PU.1, drives erythroid terminal differentiation (ETD) from the colony-forming unit–erythroid (CFU-E) progenitor stage.

With advances in single-cell RNA sequencing, a link between cell fate and cell cycle has also been suggested in the human erythroid and megakaryocyte lineages. Cell cycle genes are enriched in megakaryocytic progenitors (MKPs) and erythroid progenitors (ERPs), but MKPs more prominently express the cell cycle machinery genes CDCs and CDKs, whereas ERPs more so express TP53 (p53), MYC, and CDKN1C (p57). This differential gene expression is functionally important. Short-hairpin RNA-mediated knockdown of MYC in MEPs decreased proliferation and increased MK lineage colonies at the expense of E colonies. On the other hand, knockdown of p53 in MEPs increased cell proliferation and E specification, demonstrating that p53 and MYC are effectors in MEP fate. It was further shown that most fast-cycling MEP cells were E-specified, most slow-cycling cells were bipotent MEPs, and most cells with a medium proliferation speed were MK-specified. Further genetic and pharmacologic studies demonstrated that acceleration of cell cycle promoted E specification, while inhibition of cell cycling promotes MK specification, showing that cell cycling itself affects MEP fate decisions (Lu et al., 2018; Scanlon et al., 2022) (Figure 1).

The Socolovsky group demonstrated a link between cell cycle and erythroid differentiation (Pop et al., 2010). During S-phase of the last colony-forming erythroid progenitor stage, upregulation of CD71, downregulation of CDK inhibitor p57^KIP2^ and PU.1, and reconfiguration of chromatin at the erythroid-specific β-globin gene locus allow for the transition from self-renewal to erythroid terminal differentiation, and this fate switch is dependent on S-phase progression. They also demonstrated that the transition to erythroid terminal differentiation coincides with S-phase shortening due to downregulation of p57^KIP2^-mediated CDK inhibition and increase in replication fork speed (Hwang et al., 2017). This provides evidence for an S phase-dependent erythroid cell fate decision and further demonstrates that cell cycle length itself affects fate decisions of hematopoietic progenitors (Figure 1).

Transcriptional regulators involved in lineage-specific gene expression can also regulate the cell cycle in hematopoietic cells. For instance, GATA1, which is known to be critical for erythroid and megakaryocytic lineage gene expression, induces cell cycle arrest in G_1_ phase during erythroblast maturation (Rylski et al., 2003). GATA1 increases expression of Cdk inhibitors, while repressing Cyclin D2 and Cdk6, which control the G_1_/S transition (Rylski et al., 2003). GATA1 also regulates Cdc6, a replication-licensing factor, in murine erythroid and megakaryocytic cells (Fernández-Morales et al., 2012). Interestingly, once lineages are specified, several studies established the concept of mitotic bookmarking factors, whereby regulatory elements of lineage specification are bound to chromatin during mitosis in order to properly reactivate lineage specifying genes after mitosis in order to maintain cell fate (Festuccia et al., 2017). For instance, during mitosis, GATA1 remains tethered to chromatin in a subset of its target genes and this promotes rapid reactivation of these genes (Kadauke et al., 2012). In hematopoietic differentiation, other lineage-specific factors in addition to GATA1 may also act as mitotic bookmarking factors to control gene expression and cell fate, and this requires further investigation.

## Cell cycle length and division history stratifies HSC activity

**FIGURE 2:**
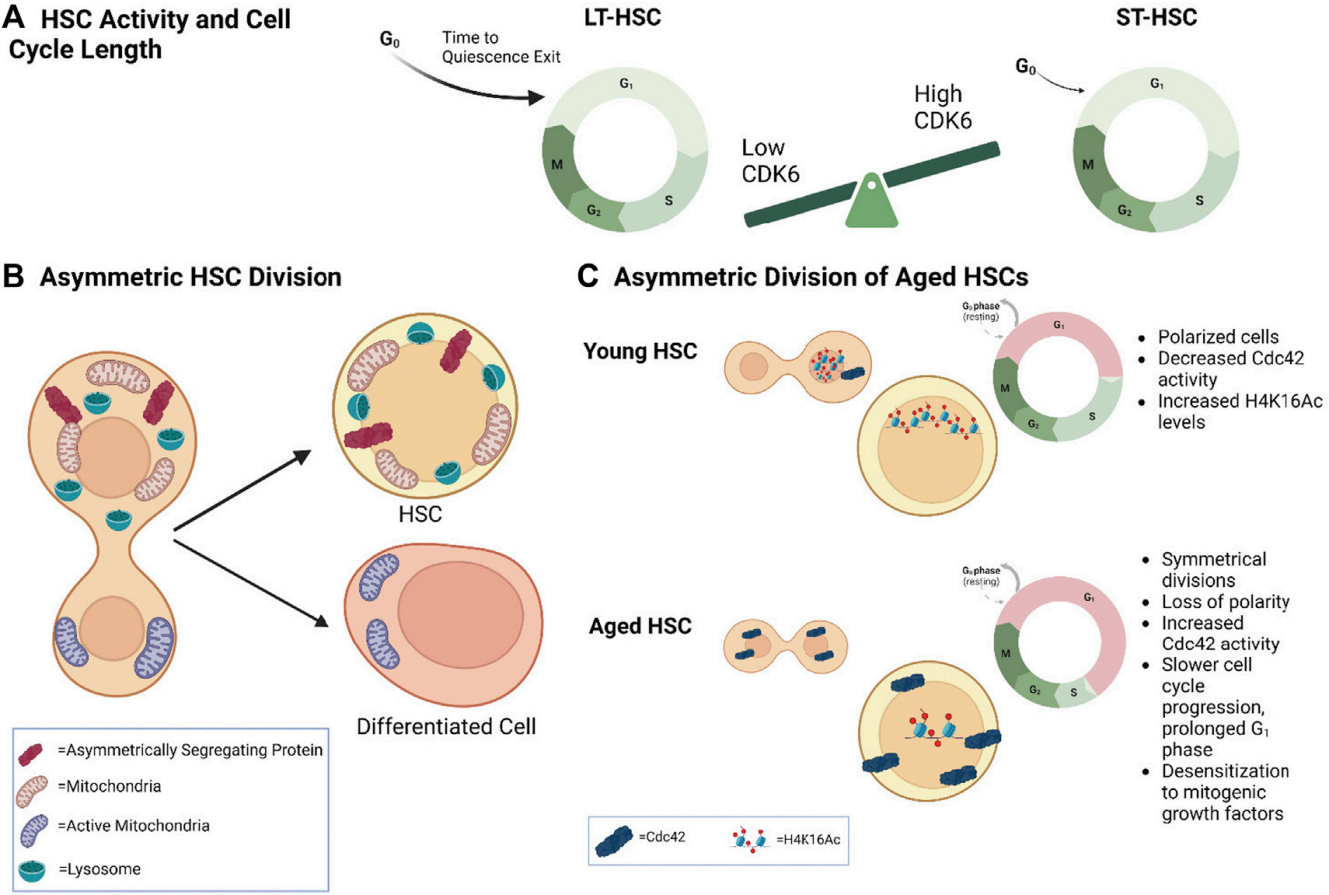
Cell Cycle Length and Asymmetric Division in HSC Fate **(A)** Model demonstrating that differential CDK6 protein levels in long–term (LT) *versus* short-term (ST)-HSCs regulates time to cell-cycle entry upon activation. **(B)** Model depicting asymmetric distribution of proteins (e.g., Ap2a2) and organelles to daughter cells and their association with different fates. Inheritance of more lysosomes correlates with higher HSC activity, while fewer lysosomes and more active mitochondria correlates with differentiation. **(C)** Model demonstrating asymmetric division in young *versus* aged HSCs. Young HSCs are more polarized and undergo more asymmetric divisions. They also contain decreased Cdc42 activity and increased H4K16 levels and activity. Aged HSCs undergo more symmetrical divisions with a loss of polarity. They have increased Cdc42 activity. Aged HSCs also have a prolonged G_1_ phase and slower cell cycle progression, as well as desensitization to mitogenic growth factors.

HSCs are mostly quiescent. They divide from time-to-time and generate progeny that will either retain stemness properties and return to dormancy and/or continue dividing and commit to differentiation in order to sustain blood cell production throughout life. These decisions are inherently linked to the cell cycle. Studies have shown that HSC activity is linked to both the duration and frequency of cycling, as well as to regulatory circuits that are present during active cycling. HSCs have been stratified by their cell cycle activity decades ago (Suda et al., 1983; Pietrzyk et al., 1985; Morrison and Weissman, 1994; Bradford et al., 1997; Uchida et al., 2003). Numerous studies have made the observation that HSC repopulation potential (LT-HSC; IT-HSC, ST-HSC) is inversely correlated with the depth of quiescence, cell cycle length or time-to-cell cycle entry, and frequency of cell divisions (Cheshier et al., 1999; Passegué et al., 2005; Wilson et al., 2008; Foudi et al., 2009; Benveniste et al., 2010; Copley et al., 2012; Oguro et al., 2013; Qiu et al., 2014). HSCs with long-term repopulation potential are deeply quiescent, have a slow division kinetic, and divide infrequently, whereas the depth of quiescence and time to cell cycle entry progressively decrease in HSCs with intermediate and short-term repopulation potential, respectively, which is associated with an increase in division frequency. The timing of exit from quiescence is regulated by the complex CDK6/cyclin D. In the human system, short-term-HSCs (defined as Lin- CD34^+^ CD38^−^ CD45RA^−^ CD90^−^ CD49f^−^), are equally quiescent as LT-HSCs (Lin- CD34^+^ CD38^−^ CD45RA^−^ CD90^+^ CD49f^+^), but contain higher CDK6 protein levels that permit faster cell cycle entry upon mitogenic stimulation, providing a molecular link between HSC activity and cell cycle length (Laurenti et al., 2015) (Figure 2A). Interestingly, enforced CDK6 expression in LT-HSCs shortens quiescence exit but still confers competitive advantage without impacting exhaustion in *in vitro* culture (Laurenti et al., 2015). Likewise, the G_1_ phase has been implicated in the regulation of human CD34^+^ HSPC fate with the finding that elevated levels of cell cycle regulator complex CCND1–CDK4 promoted G_0_ to G_1_ transition and shortened the G_1_ cell cycle phase. Overexpression of this complex in HSPCs also led to enhanced engraftment *in vivo* (Mende et al., 2015). In the mouse system, label-retention studies using a doxycycline-inducible H2B GFP label-retaining model where H2B GFP gets diluted with accrue divisions (Wilson et al., 2008; Qiu et al., 2014), have definitively demonstrated that the most dormant HSCs–i.e., cells that retain the label and can be defined as LSK-CD48^−^CD150^+^CD34^−^, have the highest and most durable repopulation potential. These cells also have a longer time-to-cell cycle entry. In the human system, the Dick group identified a subset of human LT-HSCs (CD90^+^CD49f^+^) with a latent phenotype (Kaufmann et al., 2021). These HSCs were deeply quiescent, had a prolonged time to cell cycle entry, and were characterized by low expression of CD112. CD112^Lo^ HSCs demonstrated latency in their reconstitution kinetics but still retain self-renewal and long-term reconstitution (Kaufmann et al., 2021). The link between cell cycle and HSC activity evolves with prior division history. With accrued division, HSC activity declines. The label-retention studies show that the dilution of the label is widely heterogeneous; demonstrated to represent accrued divisions, such that HSCs can be separated based on their divisional history. These studies have demonstrated that HSC activity declines with each round of division (Qiu et al., 2014; Bernitz et al., 2016). The findings supporting that HSCs divide into gradually less functional progeny have two major implications. One is that HSC self-renewal capacity is much more limited than previously thought. Second is that HSCs may have divisional memory. Studies by the Moore group suggested that HSCs are able to count and remember their divisions where they show that a population of H2B GFP label-retaining HSCs (LSK CD48^−^Flk2^−^CD150^+^) undergo four asynchronous divisions prior to becoming dormant (Bernitz et al., 2016). The label-retention studies have also shown that with increased divisional history in HSCs (LSK CD48^−^CD150^+^CD135^−^CD34^−^), there is a decreased proportion of G_0_ cells and differential expression of cell cycle pathway genes including genes encoding Cyclins and Cdks (Qiu et al., 2014). In addition, cell-cycle associated transcription factors and lineage genes are differentially upregulated in LSK CD48^−^Flk2^−^CD150^+^ HSCs with a high divisional history, which suggests that cell divisions themselves may drive lineage priming (Jeffrey et al., 2020). Other studies using single cell RNA-seq have shown that HSC priming is associated with cell cycle entry (Weinreb et al., 2020). Hence, dormancy preserves HSC activity by preventing premature exhaustion but once HSCs divide, they are programmed for differentiation or exhaustion. A model where HSCs replicate into gradually less functional progeny has been shown to occur under mild inflammatory conditions (Qiu et al., 2014; Bernitz et al., 2016; Bogeska et al., 2022). Various acute or chronic inflammatory stress have been shown to activate HSC into cycle and cause HSC exhaustion (King and Goodell, 2011). Interestingly, the negative impact of inflammation on HSC functions appears to be dependent on replicative stress. Highly-enriched LT-HSCs (LSK CD48^−^Flk2^−^CD150^+^) that maintained quiescence under repeated polyIC challenges maintained their functions. Exposure to the pro-inflammatory cytokine interleukin (IL)-1 can repress cell cycle and protein synthesis genes to maintain HSC functions (Chavez et al., 2021). These findings support that inflammation has the ability to induce HSC exit from quiescence, which contributes to their functional decline, but if HSCs remain quiescent they can repress cell cycle genes to remain protected from inflammatory stress. It will be important to know whether inflammation-driven HSC exhaustion is solely due to its effect on HSC activation into cycle, or whether it also alters HSC fate decisions during cell division, and whether different HSC subsets have distinct responses to inflammation.

## HSC fate decisions and asymmetric divisions

How HSC fate and the cell cycle are coordinated is complex and still poorly understood; but requires examining HSCs when they are actively cycling. Quiescent HSCs are metabolically inactive. They have low transcriptional and protein synthesis activities. They can rely on glycolysis and fatty acid oxidation for their energy needs (Ito et al., 2012; Takubo and Suda, 2012; Hsu and Qu, 2013; Qiu et al., 2014; Ito et al., 2016), but also on glutamine, retinoic acid and lysosomal functions (Liang et al., 2020; Sommerkamp et al., 2020; Schonberger et al., 2022). In contrast, when HSCs are activated into cycle, there is a necessary increase in mitochondrial activity and anabolic activity to support cell division (Zhu and Thompson, 2019) and commitment to differentiation (Ito et al., 2016; Ho et al., 2017; Hinge et al., 2020) This includes augmented transcription, translation, mitochondrial activity, glycolysis, and aspartate use (Ito et al., 2012; Ito et al., 2016; Karigane et al., 2016; Dong et al., 2021; Qi et al., 2021). HSCs also activate stress signaling pathways such as TGFβ, p38 MAPK, and ROS (Ito et al., 2006; Hinge et al., 2017). These pathways do not merely facilitate cell cycle progression, they also contribute to HSC fate decisions. Studies at the single cell level have demonstrated that growth factors can affect the ability of HSCs to execute self-renewal division *in vitro*. In elegant studies, Kent et al. evaluated the responses of single HSCs isolated by Rhodamine-123 (Rho) dye exclusion and EPCR expression to varying doses of Stem Cell Factor (SCF) *in vitro*. They show that the cell cycle kinetic of HSCs was unchanged by exposure to different SCF concentrations, but that HSC integrity was sustained only in the high SCF concentration when cultured for more than 8 h. SCF signaling was shown to control expression of transcription factors in HSCs while traversing the cell cycle, demonstrating that external cues control HSC fate during cell cycle progression (Kent et al., 2008).

Studies examining the process of asymmetric division have shed light on the regulation of HSC fate decisions. Hematopoietic stem cells can divide either symmetrically generating two stem cells or two progenitors, or asymmetrically, where one daughter cell can differentiate and the other retains stem cell potential. This process can be examined using the paired-daughter cell assay in which single HSCs are let to divide *in vitro*. The daughter cells generated by a single HSC are then physically separated to determine the lineage potential of each individual daughter cell. In this assay, human and mouse HSCs can generate daughter cells with equal or distinct functional behaviors in their ability to proliferate and generate multiple lineages, as assessed both *in vitro* and *in vivo* (Ema et al., 2000; Takano et al., 2004; Giebel et al., 2006). Understanding how this process is regulated has been challenging, as it requires examining HSCs at the single cell level. Plus, HSCs remain a functional entity. HSCs are retrospectively identified by their ability to produce all blood lineages for at least 4 months when transplanted in a lethally irradiated mouse. Assessments of HSC state are highly dependent on the proliferation and differentiation potential of the immediate progeny. Thus, identification of networks that regulate HSC fate decisions requires HSC analysis under conditions that do not depend on progenitor proliferation and differentiation to exclude that differences in HSC identity could be due to differences in output of daughter cell development.

Initial studies reported the unequal segregation of a number of factors, including CD53, CD62L/L-selectin, CD63/lamp-3, and CD71/transferrin receptor during human CD34^+^ HSPC division (Beckmann et al., 2007). More recent studies have now uncovered several factors that can influence the outcome of asymmetric division, including signaling and metabolic pathways, providing evidence that HSC fate decisions are linked to the cell cycle. Evidence of regulators of asymmetric cell division was shown with the asymmetrically-segregating endocytic protein Ap2a2. Ap2a2-transduced LSK-SLAM HSCs were also shown to maintain enhanced HSC activity after *in vitro* culture and secondary transplantation in the absence of increased HSC numbers, suggesting a role for Ap2a2 in HSC activity (Ting et al., 2012). Metabolic pathways can alter the fate of HSCs by changing the rate of symmetric *versus* asymmetric division. One example is the fatty acid oxidation (FAO) pathway, an important regulator of HSC functions. Inhibition of mitochondrial FAO induces HSC exhaustion by reducing LSK CD150^+^ CD48^−^ CD41^−^ Flt3^-^ CD34^−^ HSC asymmetric division and driving HSC symmetric commitment (Ito et al., 2012). NAD + -boosting agent nicotinamide riboside (NR) reduces mitochondrial activity within HSCs through increased mitochondrial clearance, leading to increased asymmetric LSK CD48^–^ CD150^+^ CD34^−^ LT-HSC divisions (Vannini et al., 2019).

Other pathways are also important for HSC fate decisions. We and others have shown the importance of Rho GTPase signaling to HSC self-renewal, migration, and adhesion (Gu et al., 2003; Yang et al., 2007; Xu et al., 2009; Zhou et al., 2013). RhoGTPases are molecular switches that regulate cytoskeleton reorganization (Etienne-Manneville and Hall, 2002). They cycle between an active GTP-bound state and an inactive GDP-bound state controlled by the opposing roles of guanine nucleotide exchange factors (GEFs), which exchange GDP for GTP, and GTPase-activating proteins (GAPs) which promote GTP hydrolysis. Our laboratory demonstrated that loss of p190-B RhoGAP enhances HSC (LSK-SLAM) self-renewal during serial transplantation without affecting HSC quiescence, survival, or multipotency (Xu et al., 2009). Using the *in vitro* paired daughter cell assay, we further demonstrated that p190B RhoGAP controls LSK-SLAM HSC asymmetric division via unequally segregating signaling molecules, including active TGF-β proteins and p38^MAPK^ pathway, to daughter cells. Elevated TGF-β activity was found to be associated with asymmetric segregation of p38^MAPK^ activity and asymmetric myeloid multilineage potential (Hinge et al., 2017). This work identified a novel role for p190-B and TGF-β-p38^MAPK^ signaling in controlling HSC fate decisions.

Another Rho GTPase, Cdc42, has been shown to contribute to HSC quiescence as well as location in the bone marrow niche (Yang et al., 2007). Cdc42 is known to interact with cell polarity protein complexes to establish asymmetry after division (Wodarz et al., 2000; Plant et al., 2003; Atwood et al., 2007). In LSK CD34^–/low^ Flk2^–^ HSCs, Cdc42 activity is asymmetrically distributed and this is associated with asymmetric division (Florian et al., 2012). Interestingly, this is associated with differential allocation of H4K16ac to daughter cells, suggesting a possible mechanistic link between asymmetric segregation of signaling molecules and asymmetric cell fate (Florian et al., 2018). This demonstrates that Rho GTPase signaling, distribution of polarity proteins, as well as epigenetic asymmetry all play a critical role in regulating daughter cell potential.

More recently it was shown by single cell time-lapse microscopy that activation of LSK-SLAM CD34-HSCs in their native niche with IFNα increases asynchronous division and asymmetrically expressed genes in paired daughter cells (Girotra et al., 2020). Single-cell RNA sequencing and transcriptomic profiling of m^6^A methyltransferase Mettl3 conditional knockout mice has also implicated RNA methylation in controlling symmetric commitment of LSK-SLAM HSCs (Cheng et al., 2019). This was shown mechanistically by m^6^A regulation of *myc* RNA stability and asymmetric segregation of Myc in daughter cells. Therefore, asymmetric segregation of factors in HSC cell division influences cell fate commitment in HSCs but this is also driven by the *in vivo* niche environment as well as epigenetic regulation (Figure 2B).

## Asymmetric HSC division and organelle distribution

In many cellular systems, organelles are asymmetrically segregated to daughter cells during cell division to drive distinct cellular fate. In yeast, the mother cell keeps damaged mitochondria to generate a ‘younger’ daughter cell, and ensure the survival of the descendant (Westermann, 2014). In contrast, human mammary epithelial-like stem cells keep the newly generated mitochondria to maintain stemness properties (Pekkai Katajisto et al., 2015). In HSCs, a functional link between asymmetric segregation of mitochondria during cell division and distinct daughter cell fate has been shown. For instance, nicotinamide riboside promotes asymmetric mitochondrial distribution in LSK-SLAM CD34^−^ LT-HSCs upon cell division and promotes LT-HSC activity (Vannini et al., 2019). The Schroeder group showed that both murine and human HSPCs can asymmetrically segregate their active mitochondria to daughter cells (Loeffler et al., 2019; Loeffler et al., 2022). Daughter cells receiving more active mitochondria also received more Myc and upregulated the transferrin receptor CD71, which was shown to predict a commitment to differentiation. Interestingly, there was an inverse correlation with lysosome inheritance. In this case, daughter cells receiving less active mitochondria inherited more lysosomes, which was associated with slow cell cycle duration and HSC activity *in vitro*. On the other hand, low levels of lysosomes were also shown to correlate with myeloid differentiation and speed of cell fate acquisition (Loeffler et al., 2019; Loeffler et al., 2022). This suggests that fate bifurcations of daughter cells are controlled by asymmetric inheritance of lysosomes (Figure 2B). This asymmetry predicts the long-term fates of the progeny of HSC daughter cells. These findings are in line with the fact that mouse and human HSC activity is associated with low mitochondrial activity but high lysosomal activity (Vannini et al., 2016; Liang et al., 2020; García-Prat et al., 2021).

The complex interplay between asymmetric HSC division, organelle inheritance, and cell cycle and fate decisions requires further investigation. How the changes in organelle and metabolic activity directly contribute to HSC fate still remain to be formally demonstrated. The exact roles of lysosomes in HSC fate decisions during activation remain unclear. One interesting possibility is the contribution of lysosomes to transitioning HSCs that are primarily in a catabolic state to a cycling anabolic state (Liang et al., 2020; García-Prat et al., 2021). Another possibility is that asymmetric inheritance of organelles could participate in divisional memory. Recently, our group showed that following transplantation, LSK-SLAM HSCs retain mitochondria that are morphologically abnormal with lower mitochondrial membrane potential (Hinge et al., 2020). Upon division of these HSCs, dysfunctional mitochondria are asymmetrically inherited by daughter cells and this is causal factor of their functional decline. Single-cell RNA sequencing demonstrated that while transplanted HSCs return to quiescence and are transcriptionally similar to non-transplanted HSCs, after activation or division, transplanted HSCs fail to upregulate pathways involved in biosynthetic and metabolic processes to support cell division (Hinge et al., 2020). These findings suggest that differences in HSC regenerative potential manifest during reactivation into cell cycle, and that mitochondria transfer information to daughter cells that will alter their fate, perhaps to remember their division ancestry and ensure the exhaustion of the HSC pool.

## Linking chromatin accessibility to cell fate in HSCs

How fate choices are regulated at the molecular level and how it is coordinated with the cell cycle are still poorly understood but likely involve chromatin remodeling and accessibility. Numerous studies have demonstrated the importance of epigenetic regulation in HSC fate decisions to self-renew or commit to differentiation through post-translational modifications of histones. Among those, histone variant H3.3, active H3K4me3, as well as bivalent H3K4me3–H3K27me3 marks are all critical for proper maintenance of HSC functions and balance between self-renewal and differentiation (McKittrick et al., 2004; Banaszynski et al., 2013; Guo et al., 2022). This is controlled by epigenetic regulators, such as Atf7ip, and Setdb1 methyltransferase, which contributes to CD41:GFP+ and CD34^+^ HSPC stemness in zebrafish and humans, respectively, by depositing H3K9me3 (Wu et al., 2023). Additionally, the histone acetyltransferase HBO1 (KAT7) has been shown to promote H3K14Ac in LSK cells at genes essential for quiescence and self-renewal in HSCs, such as Tie2, Gata2, and Gfi1b (Yang et al., 2022). Single cell barcoding experiments combined with scRNA-seq have allowed for the study of the transcriptomes, chromatin landscape, and developmental trajectories of HSCs despite their heterogeneity. These studies showed that lineage priming occurs during HSC cell cycle activation and that chromatin remodeling and accessibility occurs prior to transcriptional cellular identity and drives the differentiation process (Weinreb et al., 2020). Other studies in human HSPCs demonstrated that transcriptionally-similar HSPCs differ in chromatin landscape and accessibility but have distinct lineage-specific transcription factor activity and can adopt different fates (Ranzoni et al., 2021). It was also recently suggested that initial nonspecific global genome decompaction allows for a variable multi-lineage-primed phase followed by selective transcription to regulate key lineage gene transcription in human CD34^+^ cord blood-derived cells (Parmentier et al., 2022). These findings imply that the programs that regulate specific cell fates may be primed on the chromatin level prior to commitment to a specific lineage (Martin et al., 2021).

Several studies have provided evidence linking cell cycle and epigenetic modifications. Using the Mx1-Cre/loxP system, it was demonstrated that loss of trithorax protein ASH2l, which complexes with the H3K4 methyltransferase KMT2, leads to deregulation of mitosis-associated genes, particularly those associated with G_2_/M, and accumulation of lin^−^Sca1^+^Kit^+^ (LSK) cells in the G_2_ phase, unable to proliferate and differentiate (Lüscher-Firzlaff et al., 2019). Therefore, changes in epigenetic regulation not only alter HSC differentiation, but also regulate progression through the cell cycle itself. A recent preprint has implicated the chromatin-associated Sin3B protein in regulating cell cycle progression and differentiation in LSK-SLAM Flk2- HSCs (bioRxiv doi: https://doi.org/10.1101/2023.01.23.525185) (Alexander et al., 2023). Sin3B was shown to be necessary for HSC commitment to differentiation, and was also found to restrict progression along G_1_ phase in LT-HSCs. Loss of Sin3B was also shown to alter the chromatin state of CTCF-bound sites that are important for lineage priming in LT-HSCs. This suggests Sin3B also permits a permissive environment for differentiation and implicates the G_1_ phase of the cell cycle in regulating HSC lineage commitment through the modulation of chromatin (Alexander et al., 2023).

In a model of HSC regeneration following myeloablative chemotherapy, a recent study found that chromatin reorganization and increased transcription of transposable elements (TEs) occurs in LSK-SLAM HSCs during the activation phase (Clapes et al., 2021). TEs can bind and activate MDA5, an innate immune receptor which upon activation induces inflammatory signaling after chemotherapy. This pathway is necessary for HSC cell cycle entry, and better long-term repopulation capacity. This demonstrates the importance of TEs for HSC activation into cell cycle. Interestingly, in human HSCs, the 3-dimensional organization of the genome changes with cell cycle entry. Single-cell and bulk ATAC-seq revealed enrichment of CCCTC-binding factor (CTCF) binding, which is involved in chromatin looping and 3D genome reorganization, in activated human HSPCs compared to more dormant LT-HSCs (CD34^+^/CD38^−^/CD45RA^−^ cells) (Naoya Takayama et al., 2021). The authors also demonstrated that CTCF is required to repress genes involved in stemness and dormancy through 3D chromatin interactions, thus promoting activation of LT-HSCs. The phenomenon of bookmarking needs to be examined in the context of HSC fate decisions. It is possible that HSC functional decline through division is due to lack of stemness gene bookmarking during divisions.

## HSC self-renewal *ex vivo*

The knowledge on linking cell divisions to HSC fate and functions is important for clinical applications. Given their extensive regeneration potential, HSCs have been used for clinical HSC transplantation (HSCT) for decades. Yet, the success of HSCT depends on HSC numbers and their regenerative capacity, which has been a great limitation. Many studies have attempted to expand fully functioning HSCs *ex vivo* with limited results. Failure of numerous HSC expansion protocols arises from gaps in knowledge of the fundamental mechanisms linking HSC fate decisions to cell division. A recent study identified a culture system that allowed for the long-term *ex vivo* expansion of functional HSCs, utilizing polyvinyl alcohol (PVA) in place of serum albumin, and found that high levels of thrombopoietin (TPO) synergized with low levels of stem-cell factor (SCF) and fibronectin to promote LSK CD150^+^ CD34^−^ HSC self-renewal (Wilkinson et al., 2019). These culture conditions afforded between 236 and 899-fold expansion of functional HSCs with multilineage repopulation potential, over a period of 1 month. Importantly, these cultures also engrafted in non-irradiated recipients, which has important clinical implications for HSC-based therapies (Wilkinson et al., 2019). More recently, the authors also examined the compatibility of different PVA types in supporting human and mouse HSC activity in *ex vivo* culture. They discovered that PVA supports human cord blood CD34^+^ HSPC multilineage reconstitution in both irradiated and non-irradiated recipients as well, but that different PVA types may differentially support HSC populations such as human vs. mouse HSC and LT-vs ST-HSC (Sudo et al., 2021). This study indicates that functional HSCs can be maintained and even expanded under proliferative conditions *ex vivo*, implying that HSCs can execute self-renewing divisions *ex vivo*. The mechanism behind this HSC expansion is unclear. More information is needed on whether it is due to controlling cell cycle length to enable fate decisions toward self-renewal instead of differentiation, or changes in chromatin architecture or metabolism of these cultured cells.

## HSC cell cycling rate evolves with aging

It is well understood that as an organism ages, the characteristics of the HSC compartment change. The HSC pool expands phenotypically, but HSC self-renewal and regenerative functions decline. Old HSCs lose their ability to balance lineage output, and exhibit myeloid skewing as well as an expansion of myeloid-restricted repopulating cells (de Haan and Lazare, 2018). This is driven by various cell-intrinsic mechanisms such as DNA damage and impaired autophagy and mitochondrial activity, as well as extrinsic changes in the microenvironment (Yamamoto et al., 2018). Interestingly, the cell cycle of aged HSCs also changes. During development, there is a postnatal switch where the HSC population transitions from a rapidly cycling state to a quiescent state (Bowie et al., 2006). Label-retention experiments in older mice suggest that aged HSC progressively lengthen periods between divisions (Bernitz et al., 2016). Single-cell RNA sequencing comparing young and aged LSK-SLAM HSCs revealed a lower frequency of cells in the G_1_ phase among old compared with young HSCs (Kowalczyk et al., 2015). Another study that analyzed a large number of cells at a deeper resolution and used LSK-SLAM Flt3^−^ to define LT-HSCs, suggested that aged HSCs have a delay in differentiation caused by cell cycle arrest and imbalance of cell cycle regulators (Hérault et al., 2021). Interestingly, the Nakauchi group reported a subset of HSCs in the aged LSK CD150^+^ CD41^+^ CD34^−^ compartment that exhibited myeloid-restricted repopulating activity in primary recipients acquired lymphoid potential upon secondary transplantation, suggesting the existence of a population of “latent-balanced HSCs” in aged mice (Yamamoto et al., 2018).

In humans, aging is also accompanied by an accumulation of phenotypic HSCs that have decreased repopulation activity (Rossi et al., 2005). A recent single-cell analysis of human HSC-enriched cells (CD34^+^CD38^−^CD45RA^−^CD90^+^CD49f^+^) from cord blood, adult bone marrow, and mobilized peripheral blood revealed that increasing donor age was associated with progressively delayed cell cycle progression and prolonged G_1_ phase, as well as desensitization to mitogenic stimulation by extrinsic growth factors (Hammond et al., 2023) (Figure 2C). Interestingly, no differences in lineage output were found in this study, though the authors discuss that appropriate stimulation and beneficial transplant conditions may explain this discrepancy. These studies suggest that HSC activity and the cell cycle are coordinated, evolve with time, and contribute to the aged HSC phenotype.

In elegant and difficult studies, the Geiger group used paired-daughter cell assays in which the daughter cells of a single cell division are physically separated and individually transplanted into lethally irradiated mice to show that old HSCs execute symmetric self-renewing divisions more frequently than young HSCs. This symmetric mode of division is associated with symmetric distribution of the RhoGTPase Cdc42 and tubulin in old LSK CD34^−^Flk2^−^ HSCs (Florian et al., 2018). Interestingly, old HSCs exhibit higher Cdc42 activity than young HSCs (Florian et al., 2012) (Figure 2C). Pharmacological inhibition of Cdc42 activity is remarkably sufficient to rejuvenate the repopulation potential of old HSCs, including their lymphoid potential. Inhibiting Cdc42 activity is also sufficient to restore asymmetric organization of old HSCs. In this case, Cdc42 appears to control the level of H4K16Ac in HSC as well as its distribution in the nucleus (Florian et al., 2012). These data indicate that cell fate decisions are altered during aging. It will be interesting to fully understand the link between cell cycle speed, mode of cell division, and lineage specification, particularly myeloid bias, in young and aged HSCs.

## Concluding remarks

**FIGURE 3:**
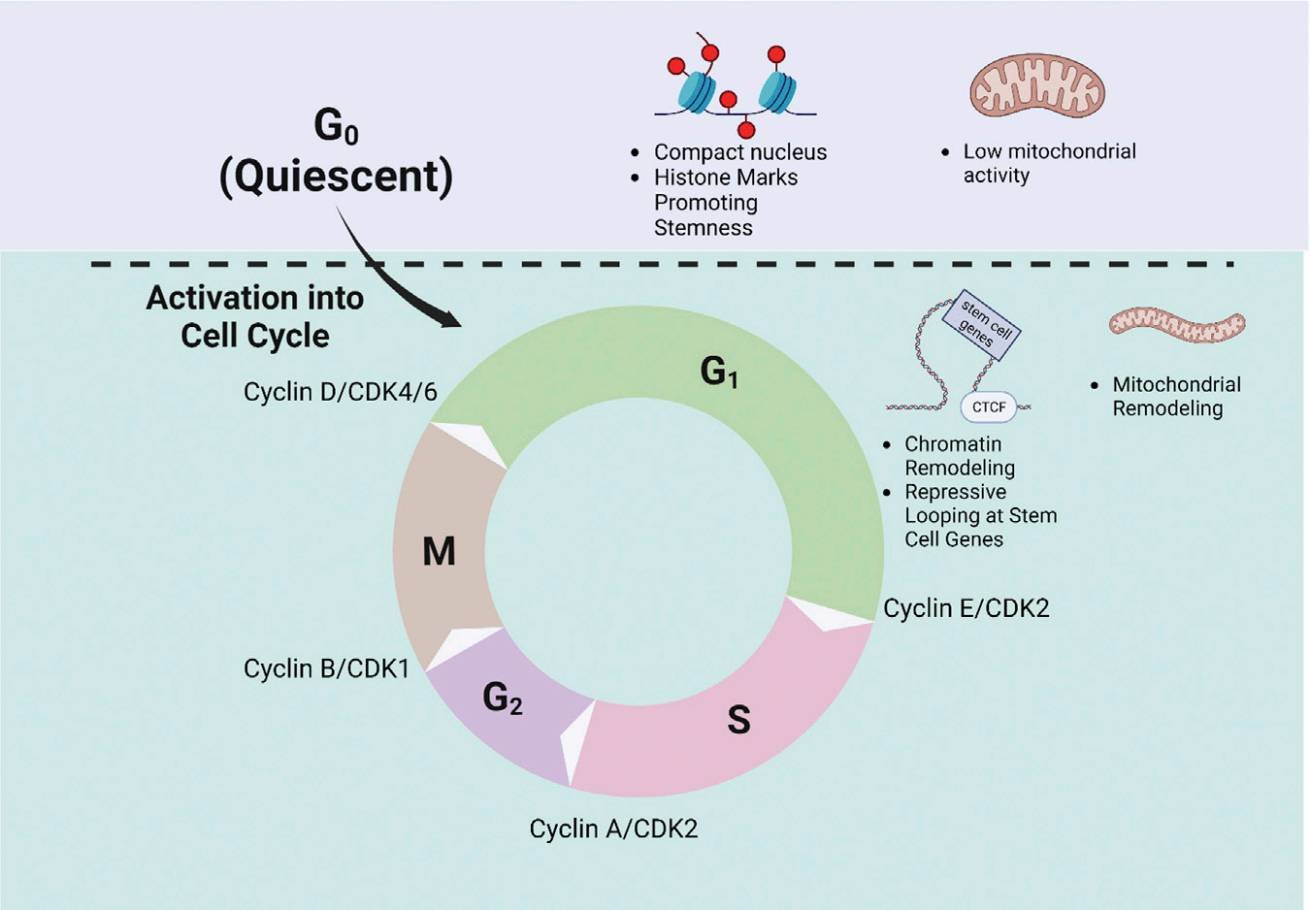
Regulation of Cell Cycle Progression and HSC fate. Schematic demonstrating the phases of the cell cycle and exit from quiescence is shown. Quiescent HSCs are shown to have a compact nucleus and an epigenetic landscape favoring stemness, as well as low mitochondrial activity. Upon cell cycle entry, HSCs undergo chromatin and mitochondrial remodeling. The regulatory Cyclin-Cdk complexes at each checkpoint are also depicted.

Substantial progress has been made in our understanding of HSC fate. Accumulating evidence indicates that HSC fate and cell cycle progression are coupled in several ways (Figure 3). Cell cycle seems to provide a permissive state for lineage priming perhaps via chromatin accessibility–enabling HSCs to properly balance differentiation and stemness characteristics. A number of questions remain. What controls chromatin remodeling, how it shapes HSC fate, and how exactly fate decisions and cell cycle are coordinated remain to be understood. Metabolism may be central to linking HSC fate to the cell cycle. The cell cycle is accompanied by substantial metabolic reprogramming during which metabolite intermediates that are essential for epigenetic modification are produced. Mitochondrial inheritance seems to be critical for shaping the fate of daughter HSCs. Understanding how cell cycle, mitochondrial inheritance, metabolism, and chromatin remodeling are coordinated may well provide key answers to the long-standing questions of how HSC fate decisions are made. A better understanding of the drivers of HSC fate will allow for improved understanding of their function both physiologically and in the context of disease and aging.

## References

[B1] Adolfsson J. Borge O. J. Bryder D. Theilgaard-Mönch K. Astrand-Grundström I. Sitnicka E. (2001). Upregulation of Flt3 expression within the bone marrow Lin(-)Sca1(+)c-kit(+) stem cell compartment is accompanied by loss of self-renewal capacity. Immunity 15 (4), 659–669. 10.1016/s1074-7613(01)00220-5 11672547

[B2] Alexander C. Mestvirishvili T. Boccalatte F. Rugges K. David G. (2023). The Sin3B chromatin modifier restricts cell cycle progression to dictate hematopoietic stem cell differentiation. bioRxiv 2023. 10.1101/2023.01.23.525185

[B3] Atwood S. X. Chabu C. Penkert R. R. Doe C. Q. Prehoda K. E. (2007). Cdc42 acts downstream of Bazooka to regulate neuroblast polarity through Par-6 aPKC. J. Cell Sci. 120 (18), 3200–3206. 10.1242/jcs.014902 17726059 PMC1988841

[B4] Banaszynski L. A. Wen D. Dewell S. Whitcomb S. J. Lin M. Diaz N. (2013). Hira-dependent histone H3.3 deposition facilitates PRC2 recruitment at developmental loci in ES cells. Cell 155 (1), 107–120. 10.1016/j.cell.2013.08.061 24074864 PMC3838450

[B5] Beckmann J. Scheitza S. Wernet P. Fischer J. C. Giebel B. (2007). Asymmetric cell division within the human hematopoietic stem and progenitor cell compartment: identification of asymmetrically segregating proteins. Blood 109 (12), 5494–5501. 10.1182/blood-2006-11-055921 17332245

[B6] Benveniste P. Frelin C. Janmohamed S. Barbara M. Herrington R. Hyam D. (2010). Intermediate-term hematopoietic stem cells with extended but time-limited reconstitution potential. Cell Stem Cell 6 (1), 48–58. 10.1016/j.stem.2009.11.014 20074534

[B7] Bernitz J. M. Kim H. S. MacArthur B. Sieburg H. Moore K. (2016). Hematopoietic stem cells count and remember self-renewal divisions. Cell 167 (5), 1296–1309. 10.1016/j.cell.2016.10.022 27839867 PMC5115957

[B8] Blagosklonny M. V. Pardee A. B. (2002). The restriction point of the cell cycle. Cell Cycle 1 (2), 102–109. 10.4161/cc.1.2.108 12429916

[B9] Bogeska R. Mikecin A. M. Kaschutnig P. Fawaz M. Büchler-Schäff M. Le D. (2022). Inflammatory exposure drives long-lived impairment of hematopoietic stem cell self-renewal activity and accelerated aging. Cell Stem Cell 29 (8), 1273–1284 e8. 10.1016/j.stem.2022.06.012 35858618 PMC9357150

[B10] Bowie M. B. McKnight K. D. Kent D. G. McCaffrey L. Hoodless P. A. Eaves C. J. (2006). Hematopoietic stem cells proliferate until after birth and show a reversible phase-specific engraftment defect. J. Clin. Invest. 116 (10), 2808–2816. 10.1172/JCI28310 17016561 PMC1578623

[B11] Bradford G. B. Williams B. Rossi R. Bertoncello I. (1997). Quiescence, cycling, and turnover in the primitive hematopoietic stem cell compartment. Exp. Hematol. 25 (5), 445–453. 9168066

[B12] Chavez J. S. P. U. Loeffler D. Higa K. C. Hernandez G. Mills T. S. (2021). PU.1 enforces quiescence and limits hematopoietic stem cell expansion during inflammatory stress. J. Exp. Med. 218 (6), e20201169. 10.1084/jem.20201169 33857288 PMC8056754

[B13] Cheng Y. Luo H. Izzo F. Pickering B. F. Nguyen D. Myers R. (2019). m(6 A RNA methylation maintains hematopoietic stem cell identity and symmetric commitment. Cell Rep. 28 (7), 1703–1716. 10.1016/j.celrep.2019.07.032 31412241 PMC6818972

[B14] Cheshier S. H. Morrison S. J. Liao X. Weissman I. L. (1999). *In vivo* proliferation and cell cycle kinetics of long-term self-renewing hematopoietic stem cells. Proc. Natl. Acad. Sci. U. S. A. 96 (6), 3120–3125. 10.1073/pnas.96.6.3120 10077647 PMC15905

[B15] Clapes T. Polyzou A. Prater P. Sagar Morales-Hernández A. Ferrarini M. G. (2021). Chemotherapy-induced transposable elements activate MDA5 to enhance haematopoietic regeneration. Nat. Cell Biol. 23 (7), 704–717. 10.1038/s41556-021-00707-9 34253898 PMC8492473

[B16] Copley M. R. Beer P. A. Eaves C. J. (2012). Hematopoietic stem cell heterogeneity takes center stage. Cell Stem Cell 10 (6), 690–697. 10.1016/j.stem.2012.05.006 22704509

[B17] Dalton S. (2015). Linking the cell cycle to cell fate decisions. Trends Cell Biol. 25 (10), 592–600. 10.1016/j.tcb.2015.07.007 26410405 PMC4584407

[B18] de Haan G. Lazare S. S. (2018). Aging of hematopoietic stem cells. Blood 131 (5), 479–487. 10.1182/blood-2017-06-746412 29141947

[B19] Dong S. Wang Q. Kao Y. R. Diaz A. Tasset I. Kaushik S. (2021). Chaperone-mediated autophagy sustains haematopoietic stem-cell function. Nature 591 (7848), 117–123. 10.1038/s41586-020-03129-z 33442062 PMC8428053

[B20] Ema H. Takano H. Sudo K. Nakauchi H. (2000). *In vitro* self-renewal division of hematopoietic stem cells. J. Exp. Med. 192 (9), 1281–1288. 10.1084/jem.192.9.1281 11067877 PMC2193353

[B21] Etienne-Manneville S. Hall A. (2002). Rho GTPases in cell biology. Nature 420 (6916), 629–635. 10.1038/nature01148 12478284

[B22] Fernández-Morales B. Pavón L. Calés C. (2012). CDC6 expression is regulated by lineage-specific transcription factor GATA1. Cell Cycle 11 (16), 3055–3066. 10.4161/cc.21471 22871742 PMC3442916

[B23] Festuccia N. Gonzalez I. Owens N. Navarro P. (2017). Mitotic bookmarking in development and stem cells. Development 144 (20), 3633–3645. 10.1242/dev.146522 29042475

[B24] Florian M. C. Dörr K. Niebel A. Daria D. Schrezenmeier H. Rojewski M. (2012). Cdc42 activity regulates hematopoietic stem cell aging and rejuvenation. Cell Stem Cell 10 (5), 520–530. 10.1016/j.stem.2012.04.007 22560076 PMC3348626

[B25] Florian M. C. Klose M. Sacma M. Jablanovic J. Knudson L. Nattamai K. J. (2018). Aging alters the epigenetic asymmetry of HSC division. PLoS Biol. 16 (9), e2003389. 10.1371/journal.pbio.2003389 30235201 PMC6168157

[B26] Foudi A. Hochedlinger K. Van Buren D. Schindler J. W. Jaenisch R. Carey V. (2009). Analysis of histone 2B-GFP retention reveals slowly cycling hematopoietic stem cells. Nat. Biotechnol. 27 (1), 84–90. 10.1038/nbt.1517 19060879 PMC2805441

[B27] García-Prat L. Kaufmann K. B. Schneiter F. Voisin V. Murison A. Chen J. (2021). TFEB-mediated endolysosomal activity controls human hematopoietic stem cell fate. Cell Stem Cell 28 (10), 1838–1850.e10. 10.1016/j.stem.2021.07.003 34343492

[B28] Giebel B. Zhang T. Beckmann J. Spanholtz J. Wernet P. Ho A. D. (2006). Primitive human hematopoietic cells give rise to differentially specified daughter cells upon their initial cell division. Blood 107 (5), 2146–2152. 10.1182/blood-2005-08-3139 16249381

[B29] Girotra M. Trachsel V. Roch A. Lutolf M. P. (2020). *In vivo* pre-instructed HSCs robustly execute asymmetric cell divisions *in vitro* . Int. J. Mol. Sci. 21, 8225. 10.3390/ijms21218225 33153113 PMC7663432

[B30] Gu Y. Filippi M. D. Cancelas J. A. Siefring J. E. Williams E. P. Jasti A. C. (2003). Hematopoietic cell regulation by Rac1 and Rac2 guanosine triphosphatases. Science 302 (5644), 445–449. 10.1126/science.1088485 14564009

[B31] Guo P. Liu Y. Geng F. Daman A. W. Liu X. Zhong L. (2022). Histone variant H3.3 maintains adult haematopoietic stem cell homeostasis by enforcing chromatin adaptability. Nat. Cell Biol. 24 (1), 99–111. 10.1038/s41556-021-00795-7 34961794 PMC9166935

[B32] Haas S. Trumpp A. Milsom M. D. (2018). Causes and consequences of hematopoietic stem cell heterogeneity. Cell Stem Cell 22 (5), 627–638. 10.1016/j.stem.2018.04.003 29727678

[B33] Hammond C. A. Wu S. W. Wang F. MacAldaz M. E. Eaves C. J. (2023). Aging alters the cell cycle control and mitogenic signaling responses of human hematopoietic stem cells. Blood 141 (16), 1990–2002. 10.1182/blood.2022017174 36652668

[B34] Hérault L. Poplineau M. Mazuel A. Platet N. Remy É. Duprez E. (2021). Single-cell RNA-seq reveals a concomitant delay in differentiation and cell cycle of aged hematopoietic stem cells. BMC Biol. 19 (1), 19. 10.1186/s12915-021-00955-z 33526011 PMC7851934

[B35] Hinge A. He J. Bartram J. Javier J. Xu J. Fjellman E. (2020). Asymmetrically segregated mitochondria provide cellular memory of hematopoietic stem cell replicative history and drive HSC attrition. Cell Stem Cell 26 (3), 420–430. 10.1016/j.stem.2020.01.016 32059807 PMC7212526

[B36] Hinge A. Xu J. Javier J. Mose E. Kumar S. Kapur R. (2017). p190-B RhoGAP and intracellular cytokine signals balance hematopoietic stem and progenitor cell self-renewal and differentiation. Nat. Commun. 8 (1), 14382. 10.1038/ncomms14382 28176763 PMC5309857

[B37] Ho T. T. Warr M. R. Adelman E. R. Lansinger O. M. Flach J. Verovskaya E. V. (2017). Autophagy maintains the metabolism and function of young and old stem cells. Nature 543 (7644), 205–210. 10.1038/nature21388 28241143 PMC5344718

[B38] Hsu P. Qu C. K. (2013). Metabolic plasticity and hematopoietic stem cell biology. Curr. Opin. Hematol. 20 (4), 289–294. 10.1097/MOH.0b013e328360ab4d 23615055 PMC3736335

[B39] Hwang Y. Futran M. Hidalgo D. Pop R. Iyer D. R. Scully R. (2017). Global increase in replication fork speed during a p57KIP2-regulated erythroid cell fate switch. Sci. Adv. 3 (5), e1700298. 10.1126/sciadv.1700298 28560351 PMC5446218

[B40] Ito K. Carracedo A. Weiss D. Arai F. Ala U. Avigan D. E. (2012). A PML–PPAR-δ pathway for fatty acid oxidation regulates hematopoietic stem cell maintenance. Nat. Med. 18 (9), 1350–1358. 10.1038/nm.2882 22902876 PMC3566224

[B41] Ito K. Hirao A. Arai F. Takubo K. Matsuoka S. Miyamoto K. (2006). Reactive oxygen species act through p38 MAPK to limit the lifespan of hematopoietic stem cells. Nat. Med. 12 (4), 446–451. 10.1038/nm1388 16565722

[B42] Ito K. T. R. Cui J. Zimmerman S. E. Pinho S. Mizoguchi T. Arai F. (2016). Self-renewal of a purified Tie2+ hematopoietic stem cell population relies on mitochondrial clearance. Science 354 (6326), 1156–1160. 10.1126/science.aaf5530 27738012 PMC5164878

[B43] Jeffrey M. Bernitz K. R. Daniel M. G. Shcherbinin D. Yuan Y. Gomes A. (2020). Memory of divisional history directs the continuous process of primitive hematopoietic lineage commitment. Stem Cell Rep. 14, 561–574. 10.1016/j.stemcr.2020.03.005 PMC7160360 32243840

[B44] Kadauke S. Udugama M. I. Pawlicki J. M. Achtman J. C. Jain D. P. Cheng Y. (2012). Tissue-specific mitotic bookmarking by hematopoietic transcription factor GATA1. Cell 150 (4), 725–737. 10.1016/j.cell.2012.06.038 22901805 PMC3425057

[B45] Karigane D. Kobayashi H. Morikawa T. Ootomo Y. Sakai M. Nagamatsu G. (2016). p38α activates purine metabolism to initiate hematopoietic stem/progenitor cell cycling in response to stress. Cell Stem Cell 19 (2), 192–204. 10.1016/j.stem.2016.05.013 27345838

[B46] Kaufmann K. B. Zeng A. G. X. Coyaud E. Garcia-Prat L. Papalexi E. Murison A. (2021). A latent subset of human hematopoietic stem cells resists regenerative stress to preserve stemness. Nat. Immunol. 22 (6), 723–734. 10.1038/s41590-021-00925-1 33958784

[B47] Kent D. G. Copley M. R. Benz C. Wöhrer S. Dykstra B. J. (2009). Prospective isolation and molecular characterization of hematopoietic stem cells with durable self-renewal potential. Blood 113 (25), 6342–6350. 10.1182/blood-2008-12-192054 19377048

[B48] Kent D. G. Dykstra B. J. Cheyne J. Eaves C. J. (2008). Steel factor coordinately regulates the molecular signature and biologic function of hematopoietic stem cells. Blood 112 (3), 560–567. 10.1182/blood-2007-10-117820 18502833 PMC2481530

[B49] Kent D. G. Dykstra B. J. Eaves C. J. (2016). Isolation and assessment of single long-term reconstituting hematopoietic stem cells from adult mouse bone marrow. Curr. Protoc. Stem Cell Biol. 38, 4.1. 10.1002/cpsc.10 27532815

[B50] Kiel M. J. Yilmaz O. H. Iwashita T. Yilmaz O. H. Terhorst C. Morrison S. J. (2005). SLAM family receptors distinguish hematopoietic stem and progenitor cells and reveal endothelial niches for stem cells. Cell 121 (7), 1109–1121. 10.1016/j.cell.2005.05.026 15989959

[B51] King K. Y. Goodell M. A. (2011). Inflammatory modulation of HSCs: viewing the HSC as a foundation for the immune response. Nat. Rev. Immunol. 11 (10), 685–692. 10.1038/nri3062 21904387 PMC4154310

[B52] Kowalczyk M. S. Tirosh I. Heckl D. Rao T. N. Dixit A. Haas B. J. (2015). Single-cell RNA-seq reveals changes in cell cycle and differentiation programs upon aging of hematopoietic stem cells. Genome Res. 25 (12), 1860–1872. 10.1101/gr.192237.115 26430063 PMC4665007

[B53] Kueh H. Y. Champhekar A. Champhekhar A. Nutt S. L. Elowitz M. B. Rothenberg E. V. (2013). Positive feedback between PU.1 and the cell cycle controls myeloid differentiation. Science 341 (6146), 670–673. 10.1126/science.1240831 23868921 PMC3913367

[B54] Laurenti E. Frelin C. Xie S. Ferrari R. Dunant C. F. Zandi S. (2015). CDK6 levels regulate quiescence exit in human hematopoietic stem cells. Cell Stem Cell 16 (3), 302–313. 10.1016/j.stem.2015.01.017 25704240 PMC4359055

[B55] Liang R. Arif T. Kalmykova S. Kasianov A. Lin M. Menon V. (2020). Restraining lysosomal activity preserves hematopoietic stem cell quiescence and potency. Cell Stem Cell 26 (3), 359–376. 10.1016/j.stem.2020.01.013 32109377 PMC8075247

[B56] Loeffler D. Schneiter F. Wang W. Wehling A. Kull T. Lengerke C. (2022). Asymmetric organelle inheritance predicts human blood stem cell fate. Blood 139 (13), 2011–2023. 10.1182/blood.2020009778 34314497

[B57] Loeffler D. Wehling A. Schneiter F. Zhang Y. Müller-Bötticher N. Hoppe P. S. (2019). Asymmetric lysosome inheritance predicts activation of haematopoietic stem cells. Nature 573 (7774), 426–429. 10.1038/s41586-019-1531-6 31485073

[B58] Lu Y. C. Sanada C. Xavier-Ferrucio J. Wang L. Zhang P. X. Grimes H. L. (2018). The molecular signature of megakaryocyte-erythroid progenitors reveals a role for the cell cycle in fate specification. Cell Rep. 25 (11), 2083–2093. 10.1016/j.celrep.2018.10.084 30463007 PMC6336197

[B59] Lüscher-Firzlaff J. Chatain N. Kuo C. C. Braunschweig T. Bochyńska A. Ullius A. (2019). Hematopoietic stem and progenitor cell proliferation and differentiation requires the trithorax protein Ash2l. Sci. Rep. 9 (1), 8262. 10.1038/s41598-019-44720-3 31164666 PMC6547667

[B60] Ma Y. Kanakousaki K. Buttitta L. (2015). How the cell cycle impacts chromatin architecture and influences cell fate. Front. Genet. 6, 19. 10.3389/fgene.2015.00019 25691891 PMC4315090

[B61] Martin E. W. Krietsch J. Reggiardo R. E. Sousae R. Kim D. H. Forsberg E. C. (2021). Chromatin accessibility maps provide evidence of multilineage gene priming in hematopoietic stem cells. Epigenetics Chromatin 14 (1), 2. 10.1186/s13072-020-00377-1 33407811 PMC7789351

[B62] McKittrick E. Gafken P. R. Ahmad K. Henikoff S. (2004). Histone H3.3 is enriched in covalent modifications associated with active chromatin. Proc. Natl. Acad. Sci. U. S. A. 101 (6), 1525–1530. 10.1073/pnas.0308092100 14732680 PMC341768

[B63] Mende N. Kuchen E. E. Lesche M. Grinenko T. Kokkaliaris K. D. Hanenberg H. (2015). CCND1–CDK4–mediated cell cycle progression provides a competitive advantage for human hematopoietic stem cells *in vivo* . J. Exp. Med. 212 (8), 1171–1183. 10.1084/jem.20150308 26150472 PMC4516798

[B64] Morrison S. J. Weissman I. L. (1994). The long-term repopulating subset of hematopoietic stem cells is deterministic and isolatable by phenotype. Immunity 1 (8), 661–673. 10.1016/1074-7613(94)90037-x 7541305

[B65] Naoya Takayama A. M. Takayanagi S. Arlidge C. Zhou S. Garcia-Prat L. Chan-Seng-Yue M. (2021). The transition from quiescent to activated states in human hematopoietic stem cells is governed by dynamic 3D genome reorganization. Cell Stem Cell 28, 488–501.e10. 10.1016/j.stem.2020.11.001 33242413

[B66] Oguro H. Ding L. Morrison S. J. (2013). SLAM family markers resolve functionally distinct subpopulations of hematopoietic stem cells and multipotent progenitors. Cell Stem Cell 13 (1), 102–116. 10.1016/j.stem.2013.05.014 23827712 PMC3736853

[B67] Osawa M. Hanada K. Hamada H. Nakauchi H. (1996). Long-term lymphohematopoietic reconstitution by a single CD34-low/negative hematopoietic stem cell. Science 273 (5272), 242–245. 10.1126/science.273.5272.242 8662508

[B68] Parmentier R. Racine L. Moussy A. Chantalat S. Sudharshan R. Papili Gao N. (2022). Global genome decompaction leads to stochastic activation of gene expression as a first step toward fate commitment in human hematopoietic cells. PLoS Biol. 20 (10), e3001849. 10.1371/journal.pbio.3001849 36288293 PMC9604949

[B69] Passegué E. Wagers A. J. Giuriato S. Anderson W. C. Weissman I. L. (2005). Global analysis of proliferation and cell cycle gene expression in the regulation of hematopoietic stem and progenitor cell fates. J. Exp. Med. 202 (11), 1599–1611. 10.1084/jem.20050967 16330818 PMC2213324

[B70] Pauklin S. Vallier L. (2013). The cell-cycle state of stem cells determines cell fate propensity. Cell 155 (1), 135–147. 10.1016/j.cell.2013.08.031 24074866 PMC3898746

[B71] Pekkai Katajisto J. D. Chaffer C. Nalle P. Marjanovic N. Iqbal S. Zoncu R. (2015). Stem cells. Asymmetric apportioning of aged mitochondria between daughter cells is required for stemness. Science 348, 340–343. 10.1126/science.1260384 25837514 PMC4405120

[B72] Pietrzyk M. E. Priestley G. V. Wolf N. S. (1985). Normal cycling patterns of hematopoietic stem cell subpopulations: an assay using long-term *in vivo* BrdU infusion. Blood 66 (6), 1460–1462. 10.1182/blood.v66.6.1460.bloodjournal6661460 4063530

[B73] Plant P. J. Fawcett J. P. Lin D. C. C. Holdorf A. D. Binns K. Kulkarni S. (2003). A polarity complex of mPar-6 and atypical PKC binds, phosphorylates and regulates mammalian Lgl. Nat. Cell Biol. 5 (4), 301–308. 10.1038/ncb948 12629547

[B74] Pop R. Shearstone J. R. Shen Q. Liu Y. Hallstrom K. Koulnis M. (2010). A key commitment step in erythropoiesis is synchronized with the cell cycle clock through mutual inhibition between PU.1 and S-phase progression. PLoS Biol. 8 (9), e1000484. 10.1371/journal.pbio.1000484 20877475 PMC2943437

[B75] Qi L. Martin-Sandoval M. S. Merchant S. Gu W. Eckhardt M. Mathews T. P. (2021). Aspartate availability limits hematopoietic stem cell function during hematopoietic regeneration. Cell Stem Cell 28 (11), 1982–1999 e8. 10.1016/j.stem.2021.07.011 34450065 PMC8571029

[B76] Qiu J. Papatsenko D. Niu X. Schaniel C. Moore K. (2014). Divisional history and hematopoietic stem cell function during homeostasis. Stem Cell Rep. 2 (4), 473–490. 10.1016/j.stemcr.2014.01.016 PMC3986626 24749072

[B77] Ranzoni A. M. Tangherloni A. Berest I. Riva S. G. Myers B. Strzelecka P. M. (2021). Integrative single-cell RNA-seq and ATAC-seq analysis of human developmental hematopoiesis. Cell Stem Cell 28 (3), 472–487.e7. 10.1016/j.stem.2020.11.015 33352111 PMC7939551

[B78] Rossi D. J. Bryder D. Zahn J. M. Ahlenius H. Sonu R. Wagers A. J. (2005). Cell intrinsic alterations underlie hematopoietic stem cell aging. Proc. Natl. Acad. Sci. U. S. A. 102 (26), 9194–9199. 10.1073/pnas.0503280102 15967997 PMC1153718

[B79] Rylski M. Welch J. J. Chen Y. Y. Letting D. L. Diehl J. A. Chodosh L. A. (2003). GATA-1-mediated proliferation arrest during erythroid maturation. Mol. Cell Biol. 23 (14), 5031–5042. 10.1128/mcb.23.14.5031-5042.2003 12832487 PMC162202

[B80] Scanlon V. M. Thompson E. N. Lawton B. R. Kochugaeva M. Ta K. Mayday M. Y. (2022). Multiparameter analysis of timelapse imaging reveals kinetics of megakaryocytic erythroid progenitor clonal expansion and differentiation. Sci. Rep. 12 (1), 16218. 10.1038/s41598-022-19013-x 36171423 PMC9519589

[B81] Schonberger K. Obier N. Romero-Mulero M. C. Cauchy P. Mess J. Pavlovich P. V. (2022). Multilayer omics analysis reveals a non-classical retinoic acid signaling axis that regulates hematopoietic stem cell identity. Cell Stem Cell 29 (1), 131–148 e10. 10.1016/j.stem.2021.10.002 34706256 PMC9093043

[B82] Seita J. Weissman I. L. (2010). Hematopoietic stem cell: self-renewal versus differentiation. WIREs Syst. Biol. Med. 2 (6), 640–653. 10.1002/wsbm.86 PMC2950323 20890962

[B83] Singh A. M. Chappell J. Trost R. Lin L. Wang T. Tang J. (2013). Cell-cycle control of developmentally regulated transcription factors accounts for heterogeneity in human pluripotent cells. Stem Cell Rep. 1 (6), 532–544. 10.1016/j.stemcr.2013.10.009 PMC3871385 24371808

[B84] Sommerkamp P. Altamura S. Renders S. Narr A. Ladel L. Zeisberger P. (2020). Differential alternative polyadenylation landscapes mediate hematopoietic stem cell activation and regulate glutamine metabolism. Cell Stem Cell 26 (5), 722–738. 10.1016/j.stem.2020.03.003 32229311

[B85] Suda T. Suda J. Ogawa M. (1983). Single-cell origin of mouse hemopoietic colonies expressing multiple lineages in variable combinations. Proc. Natl. Acad. Sci. U. S. A. 80 (21), 6689–6693. 10.1073/pnas.80.21.6689 6579554 PMC391236

[B86] Sudo K. Yamazaki S. Wilkinson A. C. Nakauchi H. Nakamura Y. (2021). Polyvinyl alcohol hydrolysis rate and molecular weight influence human and murine HSC activity *ex vivo* . Stem Cell Res. 56, 102531. 10.1016/j.scr.2021.102531 34509158 PMC8629160

[B87] Takano H. Sudo K. Nakauchi H. (2004). Asymmetric division and lineage commitment at the level of hematopoietic stem cells: inference from differentiation in daughter cell and granddaughter cell pairs. J. Exp. Med. 199 (3), 295–302. 10.1084/jem.20030929 14744992 PMC2211802

[B88] Takubo K. Suda T. (2012). Roles of the hypoxia response system in hematopoietic and leukemic stem cells. Int. J. Hematol. 95 (5), 478–483. 10.1007/s12185-012-1071-4 22539363

[B89] Ting S. B. Deneault E. Hope K. Cellot S. Chagraoui J. Mayotte N. (2012). Asymmetric segregation and self-renewal of hematopoietic stem and progenitor cells with endocytic Ap2a2. Blood 119 (11), 2510–2522. 10.1182/blood-2011-11-393272 22174158

[B90] Uchida N. Dykstra B. Lyons K. J. Leung F. Y. K. Eaves C. J. (2003). Different *in vivo* repopulating activities of purified hematopoietic stem cells before and after being stimulated to divide *in vitro* with the same kinetics. Exp. Hematol. 31 (12), 1338–1347. 10.1016/j.exphem.2003.09.001 14662343

[B91] Vannini N. Campos V. Girotra M. Trachsel V. Rojas-Sutterlin S. Tratwal J. (2019). The NAD-booster nicotinamide riboside potently stimulates hematopoiesis through increased mitochondrial clearance. Cell Stem Cell 24 (3), 405–418. 10.1016/j.stem.2019.02.012 30849366

[B92] Vannini N. Girotra M. Naveiras O. Nikitin G. Campos V. Giger S. (2016). Specification of haematopoietic stem cell fate via modulation of mitochondrial activity. Nat. Commun. 7, 13125. 10.1038/ncomms13125 27731316 PMC5064016

[B93] Weinreb C. Rodriguez-Fraticelli A. Camargo F. D. Klein A. M. (2020). Lineage tracing on transcriptional landscapes links state to fate during differentiation. Science 367 (6479), eaaw3381. 10.1126/science.aaw3381 31974159 PMC7608074

[B94] Westermann B. (2014). Mitochondrial inheritance in yeast. BBA Bioenerg. 1837, 1039–1046. 10.1016/j.bbabio.2013.10.005 24183694

[B95] Wilkinson A. C. Ishida R. Kikuchi M. Sudo K. Morita M. Crisostomo R. V. (2019). Long-term *ex vivo* haematopoietic-stem-cell expansion allows nonconditioned transplantation. Nature 571 (7763), 117–121. 10.1038/s41586-019-1244-x 31142833 PMC7006049

[B96] Wilson A. Laurenti E. Oser G. van der Wath R. C. Blanco-Bose W. Jaworski M. (2008). Hematopoietic stem cells reversibly switch from dormancy to self-renewal during homeostasis and repair. Cell 135 (6), 1118–1129. 10.1016/j.cell.2008.10.048 19062086

[B97] Wodarz A. Ramrath A. Grimm A. Knust E. (2000). Drosophila atypical protein kinase C associates with Bazooka and controls polarity of epithelia and neuroblasts. J. Cell Biol. 150 (6), 1361–1374. 10.1083/jcb.150.6.1361 10995441 PMC2150710

[B98] Wu J. Li J. Chen K. Liu G. Zhou Y. Chen W. (2023). Atf7ip and Setdb1 interaction orchestrates the hematopoietic stem and progenitor cell state with diverse lineage differentiation. Proc. Natl. Acad. Sci. U. S. A. 120 (1), e2209062120. 10.1073/pnas.2209062120 36577070 PMC9910619

[B99] Xu H. Eleswarapu S. Geiger H. Szczur K. Daria D. Zheng Y. (2009). Loss of the Rho GTPase activating protein p190-B enhances hematopoietic stem cell engraftment potential. Blood 114 (17), 3557–3566. 10.1182/blood-2009-02-205815 19713466 PMC2766675

[B100] Yamamoto R. Morita Y. Ooehara J. Hamanaka S. Onodera M. Rudolph K. L. (2013). Clonal analysis unveils self-renewing lineage-restricted progenitors generated directly from hematopoietic stem cells. Cell 154 (5), 1112–1126. 10.1016/j.cell.2013.08.007 23993099

[B101] Yamamoto R. Wilkinson A. C. Ooehara J. Lan X. Lai C. Y. Nakauchi Y. (2018). Large-scale clonal analysis resolves aging of the mouse hematopoietic stem cell compartment. Cell Stem Cell 22 (4), 600–607. 10.1016/j.stem.2018.03.013 29625072 PMC5896201

[B102] Yang L. Wang L. Geiger H. Cancelas J. A. Mo J. Zheng Y. (2007). Rho GTPase Cdc42 coordinates hematopoietic stem cell quiescence and niche interaction in the bone marrow. Proc. Natl. Acad. Sci. U. S. A. 104 (12), 5091–5096. 10.1073/pnas.0610819104 17360364 PMC1829299

[B103] Yang Y. Kueh A. J. Grant Z. L. Abeysekera W. Garnham A. L. Wilcox S. (2022). The histone lysine acetyltransferase HBO1 (KAT7) regulates hematopoietic stem cell quiescence and self-renewal. Blood 139 (6), 845–858. 10.1182/blood.2021013954 34724565

[B104] Zhou X. Florian M. C. Arumugam P. Chen X. Cancelas J. A. Lang R. (2013). RhoA GTPase controls cytokinesis and programmed necrosis of hematopoietic progenitors. J. Exp. Med. 210 (11), 2371–2385. 10.1084/jem.20122348 24101377 PMC3804933

[B105] Zhu J. Thompson C. B. (2019). Metabolic regulation of cell growth and proliferation. Nat. Rev. Mol. Cell Biol. 20 (7), 436–450. 10.1038/s41580-019-0123-5 30976106 PMC6592760

